# Gastric volvulus associated with shrinkage of a gastrointestinal stromal tumor by neoadjuvant imatinib: a case report

**DOI:** 10.1186/s13256-022-03735-7

**Published:** 2023-01-16

**Authors:** Yoichi Sugiyama, Toshiaki Komo, Tatsuya Tazaki, Mohei Kohyama, Shinya Takahashi, Masaru Sasaki

**Affiliations:** 1 Department of Surgery, JA Hiroshima General Hospital, Hatsukaichi, Hiroshima, 738-8503 Japan; 2 Department of Surgery, Institute of Biomedical and Health Sciences, Hiroshima University, Hiroshima, 734-8551 Japan

**Keywords:** Gastric volvulus, Gastrointestinal stromal tumor, Imatinib, Neoadjuvant therapy, Case report

## Abstract

**Background:**

During neoadjuvant chemotherapy for giant gastrointestinal stromal tumors, changes in gastrointestinal stromal tumor size are rarely associated with events such as perforation and bleeding that require emergency surgery. Moreover, it is very rare for gastrointestinal stromal tumors to shrink and become mobile, resulting in gastric volvulus. Herein, we report a case of gastrointestinal stromal tumor shrinkage during neoadjuvant imatinib treatment, resulting in gastric volvulus that required surgery. To the best of our knowledge, this is the first reported occurrence of gastric volvulus during neoadjuvant imatinib treatment for a giant gastrointestinal stromal tumor.

**Case presentation:**

A 58-year-old Japanese woman who was diagnosed with a giant gastric gastrointestinal stromal tumor and administered neoadjuvant imatinib presented to our hospital with complaints of abdominal pain and retching. Enhanced computed tomography revealed that the gastrointestinal stromal tumor had shrunk and shifted in position, and the stomach had organoaxially twisted. Accordingly, the patient was diagnosed with gastric volvulus caused by a gastric gastrointestinal stromal tumor. Conservative treatment did not improve the volvulus; hence, laparotomy was performed. The tumor developed from the lesser curvature of the stomach and caused rotation of the gastric body. The local gastric wall was resected. Histopathological examination confirmed the diagnosis of gastrointestinal stromal tumor. The patient received adjuvant imatinib for 3 years and has been alive for 5 years without recurrence.

**Conclusions:**

Gastric volvulus can be caused by the laxity of the ligaments that hold the stomach and gastric ptosis or esophageal hernia and diaphragmatic hernia; therefore, gastric gastrointestinal stromal tumors rarely cause gastric volvulus. However, a risk of torsion exists if the gastrointestinal stromal tumor develops extramural to lesser curvature and attains a certain size.

## Background

Gastric volvulus is a rare disease in which part of the stomach is twisted more than 180°, resulting in luminal obstruction and blood flow disturbance. It can be elusive to diagnose. Neonates and infants often experience an acute disease course. However, in adults, it is often accompanied by lesions such as hiatal hernia and gastric tumor, and the course is relatively chronic. Gastric volvulus caused by gastrointestinal stromal tumors (GISTs) is very rare [1], and the present case is probably the first reported occurrence during neoadjuvant imatinib treatment for a giant GIST.

## Case presentation

**Fig. 1:**
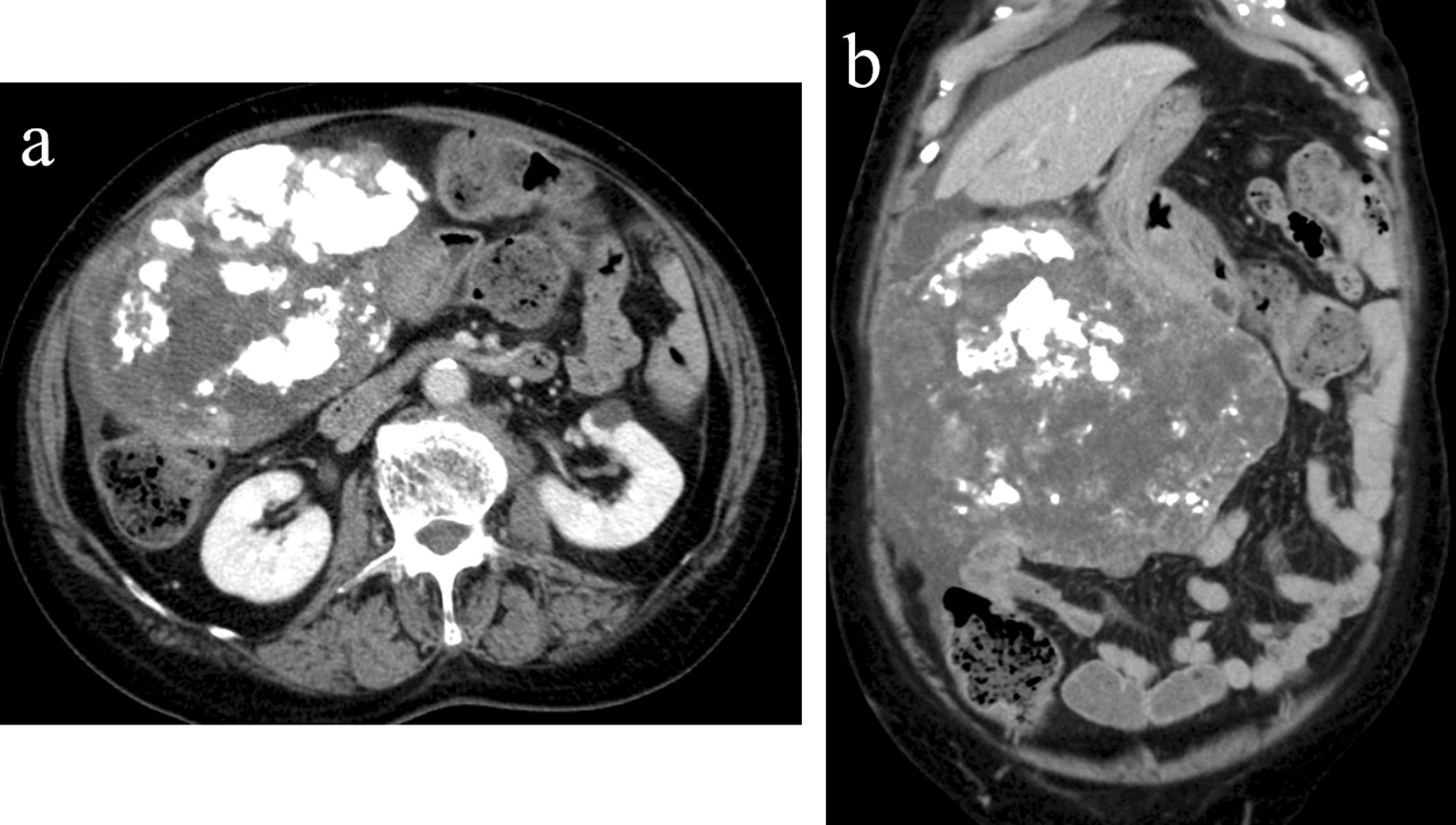
**a, b** Enhanced computed tomography reveals that the tumor had shrunk to approximately 18 cm and the stomach was twisted organoaxially

**Fig. 2:**
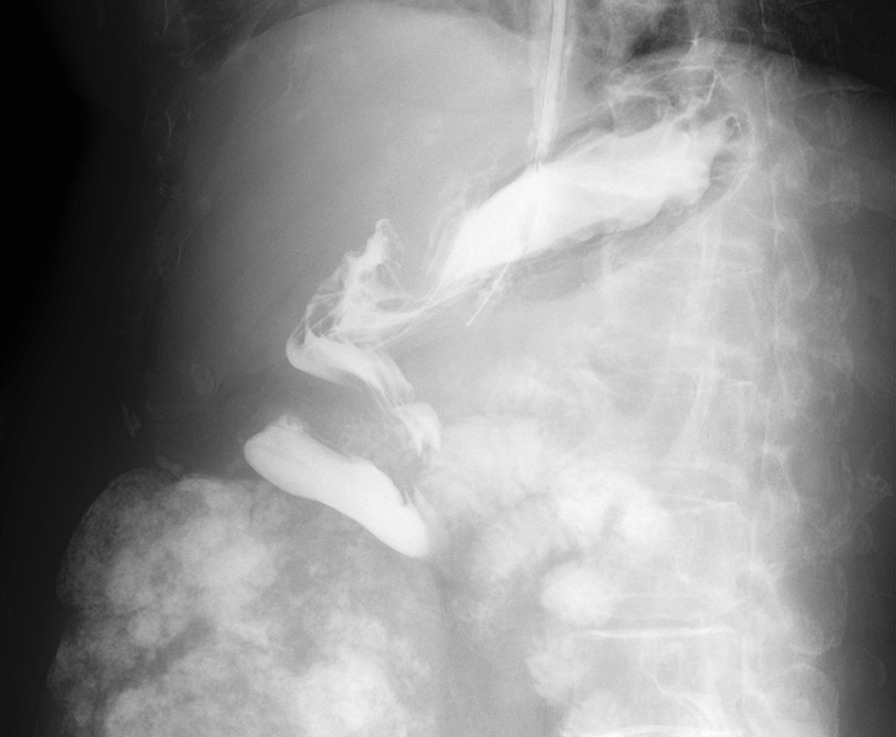
Upper gastrointestinal tract radiography reveals the rotation of the gastric body around the organoaxis

A-58-year-old Japanese woman who was diagnosed with a giant gastric GIST and administered neoadjuvant imatinib presented to the emergency department with complaints of abdominal pain and retching. Physical examination revealed abdominal distension, upper abdominal tenderness, and a moveable mass, but no rebound tenderness or guarding. Enhanced computed tomography (CT) revealed tumor shrinkage from 25 cm at the initiation of imatinib treatment to approximately 18 cm, position shift, and an organoaxially twisted stomach (Fig. 1a, b). Upper endoscopy revealed the counterclockwise twisted stomach, and the scope could not access the duodenum. Upper gastrointestinal tract contrast study revealed rotation of the gastric body around the organoaxis (Fig. 2). Based on these findings, we diagnosed the patient with gastric volvulus caused by a giant gastric GIST.

**Fig. 3:**
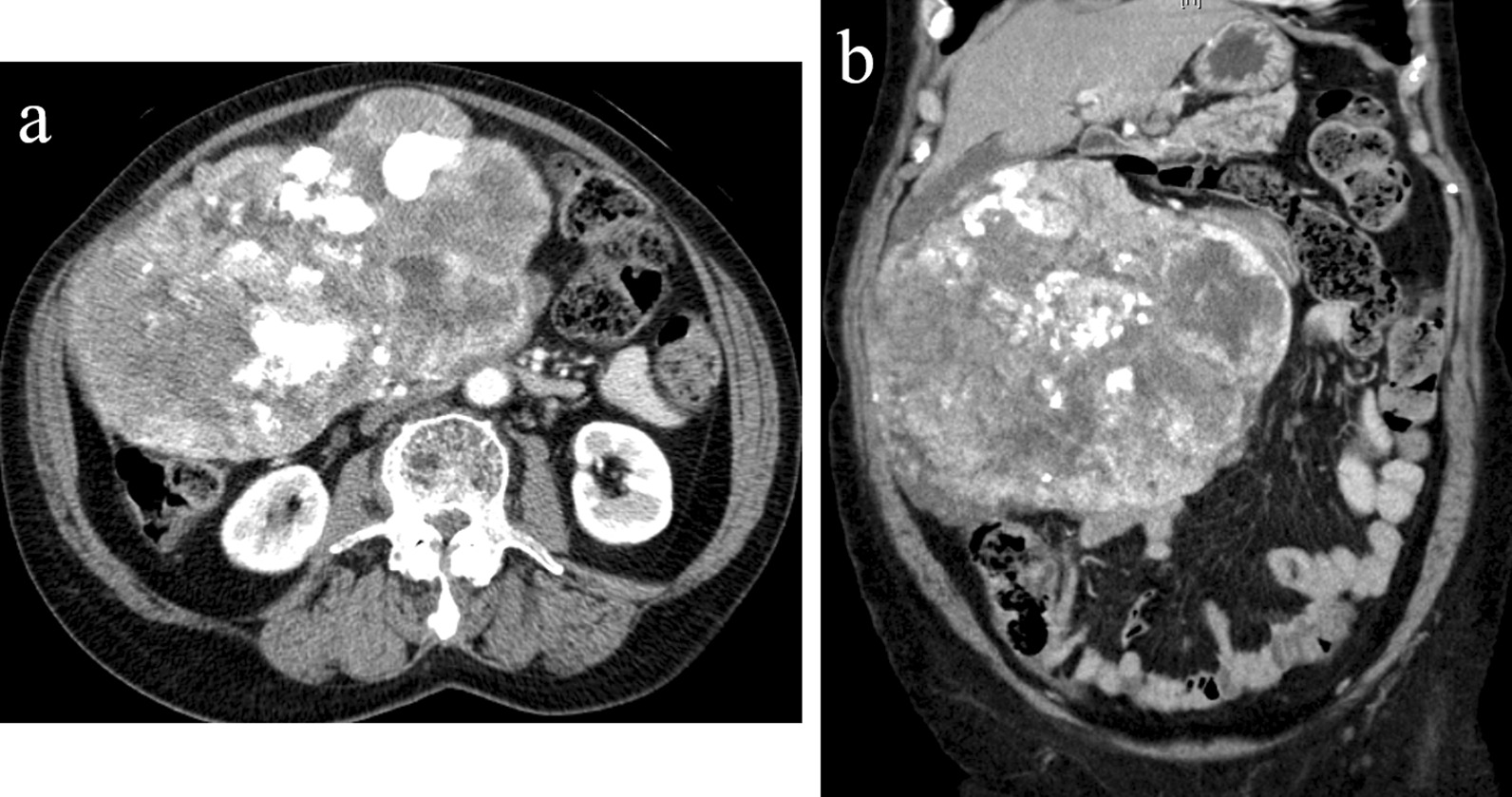
**a, b** Contrast-enhanced computed tomography showing that the tumor measured 25 × 22 cm, extended from the stomach, and abutted to the abdominal wall, liver, ascending colon, and abdominal aorta. The mass demonstrates heterogeneous contrast and internal low-density components

She had been admitted to our hospital with abdominal distention and a palpable mass 18 months earlier. The mass did not show any mobility on palpation. Enhanced CT revealed a lobulated mass measuring 25 × 22 cm, extending from the stomach, abutting the abdominal wall, liver, ascending colon, and abdominal aorta (Fig. 3a, b). Histopathological examination using endoscopic ultrasound-guided fine-needle aspiration biopsy revealed the presence of spindle cells and immunohistochemical positivity for c-kit and CD34. We diagnosed the patient with a gastric GIST; however, the tumor was bulky and had infiltrated the adjacent organs, especially the abdominal aorta; therefore, we initiated neoadjuvant imatinib treatment (400 mg/day). After 12 months of imatinib treatment, fluorodeoxyglucose-positron tomography/computed tomography (FDG-PET/CT) for response evaluation revealed metabolically inactive hypodense lesions. However, the tumor shrank slightly and showed no mobility on palpation. Eighteen months after the initiation of imatinib treatment, gastric volvulus occurred.

**Fig. 4:**
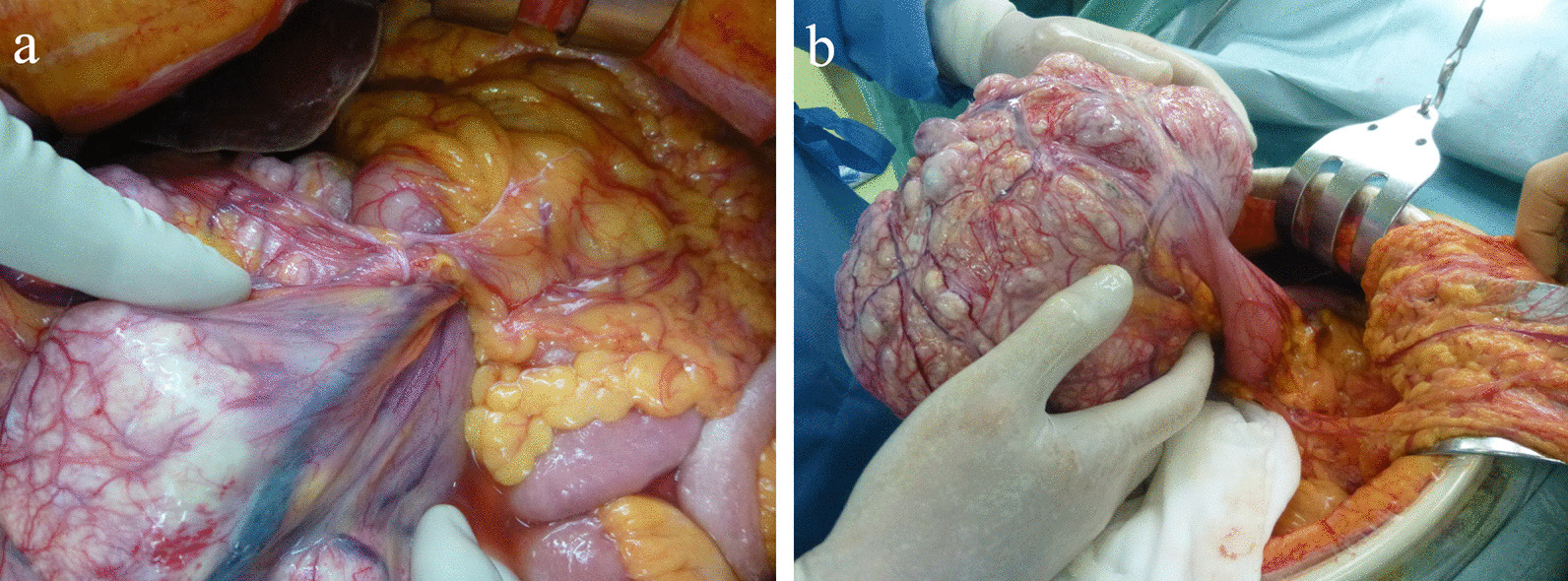
**a, b** The tumor arose from the lesser curvature of the stomach and caused the rotation of the gastric body. No other intraabdominal organs were involved. The resected tumor measured 18 × 15 × 11 cm and weighed 2000 g

**Fig. 5:**
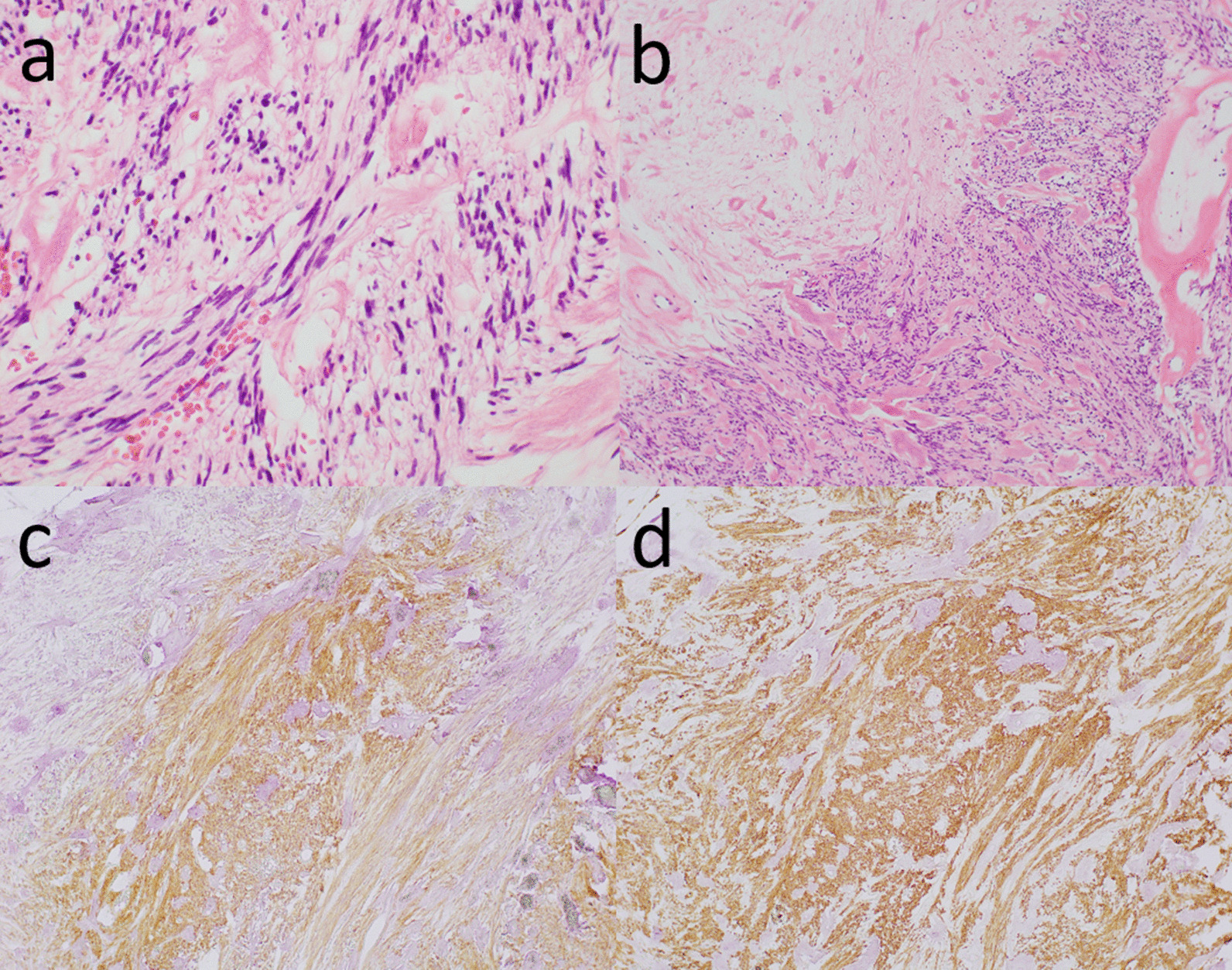
**a, b** Hematoxylin–eosin staining shows spindle-shaped mitotic tumor cells below 5/HPF and a grade 1b response to preoperative imatinib. **c, d** Immunohistochemical staining shows that the tumor is positive for KIT and CD34

For treatment, initial decompression of a nasogastric tube improved retching and abdominal distension, but did not improve the gastric volvulus. As the tumor was thought to be resectable, we decided to perform surgery using a median incision. The tumor developed from the lesser curvature of the mid-portion of the stomach and caused its rotation (Fig. 4). Other intraabdominal organs were not involved. However, the stomach did not show blood flow obstruction or necrosis, and was considered partially twisted. The main feeders of the tumor were the left and right gastric arteries. After ligation of the feeding artery, the tumor was resected along with parts of the stomach with a gross margin of 1 cm, and the gastric wall was closed with double-layer sutures. The operation time was 122 minutes and blood loss volume was 60 mL. On gross examination, the resected tumor measured 18 × 15 × 11 cm and weighed 2000 g. The postoperative course was uneventful, and the patient was discharged 9 days after the surgery. Histopathological examination confirmed the diagnosis of GIST (with KIT and CD34 positivity) with mitosis under 5/high-power field (HPF) and revealed a grade 1b response to preoperative imatinib treatment (Fig. 5a, b). Genetic analysis of the c-kit mutation revealed a mutation in exon 11, which resulted in internal tandem duplications at the 3′ end of the KIT juxtamembrane domain.

The patient received adjuvant imatinib for 3 years and has been alive for 5 years without recurrence.

## Discussion and conclusions

The most common acute symptoms of GIST are gastrointestinal bleeding, abdominal pain, and ileus. Gastric GIST symptoms depend on tumor location, size, and development form [2]. Gastrointestinal bleeding is caused by tumor growth, necrosis, and exposure to ulcers in the lumen, whereas abdominal pain is caused by tumor perforation and intraabdominal bleeding. The cause of ileus is thought to be tumor compression rather than luminal obstruction. As GISTs grow externally, there is a risk of obstruction caused by GIST-induced gastric volvulus, but it has rarely been reported in practice. To the best of knowledge, this is probably the first report of gastric volvulus during imatinib administration.

In patients with gastric volvulus, the stomach is either insufficiently fixed and/or physiologically involves some torsional mechanisms, resulting in gastric rotation. Gastric volvulus is divided into two types: organoaxial volvulus (long-axis volvulus) centered along the axis connecting the cardia and pylorus, and mesenteroaxial volvulus (short-axis volvulus) centered along the axis of the greater and lesser curvature. There is also a mixed volvulus type in which both types coexist; its causes are idiopathic or secondary, depending on the pathogenesis. Idiopathic causes include laxity of the four ligaments that hold the stomach in place and gastric ptosis, whereas secondary causes include esophageal hernia, diaphragmatic hernia, and tumors. In the idiopathic type, the stomach sometimes twists more than 360°, which may lead to hemorrhage, perforation, and necrosis due to disturbed blood flow. A surgical delay can result in progression to devastating morbidities, including gastric ischemia. Therefore, it is important to identify the disease for early diagnosis. Borchardt’s triad of symptoms, including severe epigastric distension, retching, and inability to pass a nasogastric tube, are considered typical symptoms [3]. However, symptoms vary depending on the type and degree of torsion. In our case, although abdominal pain and retching were mild, it was difficult to differentiate them from the side effects of imatinib administration or the accompanying symptoms of the tumor. However, differential diagnosis should be suspected based on clinical symptoms because imaging studies show characteristic findings. CT can show dilatation of the gastric lumen and fluid retention, and gastric volvulus can be easily diagnosed, especially if the images are reconstructed using 3D-CT.

According to Velasco *et al*. [4], 85% of gastric GISTs involve a extragastric mass and 7% grew exophytically. Therefore, a relatively large GIST may develop extramurally, and its weight may cause traction and twisting of the intestine. Then, the tumor location is important; in the case of organoaxial volvulus, the tumor must be located on the lesser curvature side. To date, there are two case reports of GIST-induced gastric volvulus, one of which was an upside-down stomach due to a hiatal hernia with GIST [5]. Upside-down stomach is an idiopathic form of organoaxial volvulus associated with a high degree of esophageal hiatal hernia, wherein the stomach prolapses into the mediastinum. This condition is one of the major forms classified by Bettex *et al*. [6]. The other case was of a GIST with extramural growth in the gastric lesser curvature, resulting in an organoaxial volvulus, as in the present case [7]. In this report, the GIST was 9 cm in diameter and weighed 224 g, which is a large and heavy tumor, as in the present case, suggesting that tumor localization, diameter, and weight are important factors for tortuosity. In our case, the GIST was initially very large and was suspected to be adherent to the surrounding organs; therefore, the GIST was immobile and did not twist. However, as the GIST shrank with imatinib treatment, the GIST became mobile and volvulous. Therefore, if a giant GIST is located externally on the lesser curvature of the gastric wall and neoadjuvant imatinib is administered for its treatment, gastric volvulus may occur once the tumor shrinks and mobility is achieved.

Gastric volvulus may be treated by decompression using a nasogastric tube or it may be corrected endoscopically in the case of the mesenteric-axial type, which has no closure of the cardiac orifice [8]. However, this approach is only for symptomatic management, and surgical treatment, including tumor resection, is necessary when traction and torsion are caused by a tumor [9]. Furthermore, surgical resection is the most effective treatment for GISTs in the absence of noncurative factors. In our case, neoadjuvant imatinib was administered to avoid complicated resection owing to the large tumor size and suspected adhesion and invasion of the surrounding organs.

Neoadjuvant imatinib for giant gastric GIST has not been established, but it is expected to shrink the tumor, prevent tumor rupture, and preserve stomach function by avoiding total gastrectomy. Kurokawa *et al*. [10] reported a phase II study of neoadjuvant imatinib treatment for giant gastric GISTs. The R0 resection rate without tumor rupture was 91%, and only 6% of patients required total gastrectomy. The response to imatinib treatment can be predicted during the treatment course using genetic typing [11]. However, if genetic analysis is not performed or kit mutations cannot be detected, imaging examinations should be performed after the first month of imatinib treatment. In particular, early evaluation using PET-CT is recommended to assess treatment response in patients treated with neoadjuvant imatinib [12]. Evaluating metabolic changes rather than morphological changes is more reliable [13]. FDG-PET/CT metabolic studies 8 weeks after initiating imatinib therapy may be useful for assessing early therapeutic effects [14]. Furthermore, > 50% reduction in standardized uptake values (SUVs) and/or an SUV of < 2.5 in the follow-up study may be a more robust criterion for the assessment of a sustained response [15]. In our case, at the first assessment after initiating imatinib treatment, the tumor did not shrink but the SUV was < 2.0. After 18 months of continued imatinib treatment, the GIST shrank and unexpectedly developed gastric volvulus; however, R0 resection was achieved. The expected time for performing resection after neoadjuvant imatinib treatment was 36–48 weeks [16] [17]; therefore, surgery should be performed once mobility is achieved and deemed possible after an earlier evaluation.

In conclusion, gastric volvulus caused by GIST is rare, but should remain part of the differential diagnosis in patients presenting with abdominal pain and retching. It should be noted that a giant GIST, especially in the lesser curvature of the stomach, may twist the stomach and cause gastric volvulus.

## Data Availability

All data will be available upon request made to the corresponding author.

## Abbreviations

GIST: Gastrointestinal stromal tumor
CT: Computed tomography
SUV: Standardized uptake value
FDG-PET: Fluorodeoxyglucose-Positron emission tomography
MRI: Magnetic resonance imaging
